# Disease detection and classification in temporal lobe epilepsy: step-wise versus simultaneous AI decision models in a multisite neuroimaging study

**DOI:** 10.1093/braincomms/fcag253

**Published:** 2026-06-29

**Authors:** Erik Kaestner, Jay Sawant, Donatello Arienzo, Kyle A Hasenstab, Ezequiel Gleichgerrcht, Taha Gholipour, Anees Abrol, Reihaneh Hassanzadeh, Sophia I Thomopoulos, Clarissa L Yasuda, Lucas Scárdua Silva, Marina K M Alvim, Patrick Moloney, Andre Altmann, Helena Martins Custodio, Ev-Christin Heide, Nishant Sinha, Alice Ballerini, Julie Absil, Sara Larivière, Kai M Schubert, Carolina Ferreira-Atuesta, Gian Marco Duma, Raphaël Christin, Elisa Barbi, Renzo Guerrini, Theodor Rüber, Tobias Bauer, Benjamin Sinclair, Jacob Bunyamin, Merran R Courtney, Meng Law, Angelo Labate, Pasquale Striano, Lucy Vivash, Terence J O’Brien, Matteo Lenge, Luca Saba, Jonathan K Kleen, Paolo Bonanni, Leigh N Sepeta, Marian Galovic, Emanuele Bartolini, Victoria Ives-Deliperi, Boris C Bernhardt, Pascal Martin, Chantal Depondt, Travis Stoub, Anna Elisabetta Vaudano, Stefano Meletti, Ruben Kuzniecky, Luis Concha, Anto I Bagić, Kathryn A Davis, Richard J Staba, Niels K N Focke, Heath Pardoe, Patricia C Dugan, Orrin Devinsky, Daniel L Drane, Zhiqiang Zhang, Antonio Gambardella, Alexandra Parashos, Fernando Cendes, Paul M Thompson, Sanjay M Sisodiya, Vince D Calhoun, Leonardo Bonilha, Carrie R McDonald

**Affiliations:** Department of Radiation Medicine and Applied Sciences, University of California, San Diego, La Jolla, CA 92037, USA; Halıcıoğlu Data Science Institute, University of California, San Diego, La Jolla, CA 92093, USA; Department of Radiation Medicine and Applied Sciences, University of California, San Diego, La Jolla, CA 92037, USA; Department of Mathematics and Statistics, San Diego State University, San Diego, CA 92182, USA; Department of Neurology, Emory University, Atlanta, GA 30322, USA; Department of Neurosciences, University of California, San Diego, La Jolla, CA 92093, USA; GSU/GATech/Emory Center for Translational Research in Neuroimaging and Data Science (TReNDS), Atlanta, GA 30303, USA; Electrical and Computer Engineering, Georgia Institute of Technology, Atlanta, GA 30332, USA; Center for Translational Research in Neuroimaging and Data Science, Georgia State University, Atlanta, GA 30303, USA; Imaging Genetics Center, Mark and Mary Stevens Neuroimaging and Informatics Institute, Keck School of Medicine, University of Southern California, Marina del Rey, CA 90292, USA; Brazilian Institute of Neuroscience and Neurotechnology (BRAINN), Campinas, SP 13083-970, Brazil; Department of Neurology, University of Campinas (UNICAMP), Campinas, SP 13083-888, Brazil; Neuroimaging Laboratory, University of Campinas (UNICAMP), Campinas, SP 13083-888, Brazil; Brazilian Institute of Neuroscience and Neurotechnology (BRAINN), Campinas, SP 13083-970, Brazil; Department of Neurology, University of Campinas (UNICAMP), Campinas, SP 13083-888, Brazil; Department of Clinical and Experimental Epilepsy, UCL Queen Square Institute of Neurology, London WC1N 3BG, UK; Dublin Neurological Institute, Mater Misericordiae University Hospital, Dublin D07 W7XF, Ireland; School of Medicine, University College Dublin, Dublin D04 V1W8, Ireland; Centre for Medical Image Computing, Department of Medical Physics and Biomedical Engineering, University College London, London WC1E 6BT, UK; Department of Clinical and Experimental Epilepsy, UCL Queen Square Institute of Neurology, London WC1N 3BG, UK; Department of Neurology, University Medical Center Göttingen, Göttingen 37075, Germany; Department of Psychiatry and Psychotherapy, University of Cologne, Cologne 50937, Germany; Department of Neurology, Perelman School of Medicine, University of Pennsylvania, Philadelphia, PA 19104, USA; Department of Biomedical, Metabolic and Neural Sciences, University of Modena and Reggio Emilia, Baggiovara 41126, Italy; Department of Radiology, CUB Erasme Hospital, Hôpital Universitaire de Bruxelles, Université Libre de Bruxelles, Brussels 1070, Belgium; Department of Medical Imaging and Radiation Sciences, Faculty of Medicine and Health Sciences, Université de Sherbrooke, Sherbrooke, QC J1H 5N4, Canada; Department of Neurology, Clinical Neuroscience Center, University Hospital and University of Zurich, Zurich 8091, Switzerland; Department of Neurology, University Hospital Zurich, Zurich 8091, Switzerland; Epilepsy and Clinical Neurophysiology Unit, Scientific Institute IRCCS E. Medea, Conegliano 31015, Italy; Department of Neurology, University of California SanFrancisco, San Francisco, CA 94158, USA; Department of Neuroscience and Human Genetics, Meyer Children’s Hospital IRCCS, Florence 50139, Italy; Department of Neuroscience and Human Genetics, Meyer Children’s Hospital IRCCS, Florence 50139, Italy; Department of Neuroscience, Pharmacology and Child Health, University of Florence, Florence 50139, Italy; Department of Neuroradiology, University Hospital Bonn, Bonn 53127, Germany; Department of Epileptology, University Hospital Bonn, Bonn 53127, Germany; German Center for Neurodegenerative Diseases (DZNE), Bonn 53127, Germany; Center for Medical Data Usability and Translation, University of Bonn, Bonn 53127, Germany; Department of Neuroradiology, University Hospital Bonn, Bonn 53127, Germany; Department of Epileptology, University Hospital Bonn, Bonn 53127, Germany; German Center for Neurodegenerative Diseases (DZNE), Bonn 53127, Germany; Department of Neuroscience, School of Translational Medicine, Alfred Health, Monash University, Melbourne, VIC 53127, Australia; Department of Neuroscience, School of Translational Medicine, Alfred Health, Monash University, Melbourne, VIC 53127, Australia; Department of Neuroscience, School of Translational Medicine, Alfred Health, Monash University, Melbourne, VIC 53127, Australia; Department of Neurology, Alfred Health, Melbourne, VIC 3004, Australia; Department of Neuroscience, School of Translational Medicine, Alfred Health, Monash University, Melbourne, VIC 53127, Australia; Neurophysiopathology and Movement Disorders Clinic, University of Messina, Messina 98125, Italy; IRCCS G. Gaslini, Full Member of Epicare, Genova 16147, Italy; Department of Neurosciences, Rehabilitation, Ophthalmology, Genetics, Maternal and Child Health, University of Genova, Genova 16132, Italy; Department of Neuroscience, School of Translational Medicine, Alfred Health, Monash University, Melbourne, VIC 53127, Australia; Department of Neurology, Alfred Health, Melbourne, VIC 3004, Australia; Department of Neuroscience, School of Translational Medicine, Alfred Health, Monash University, Melbourne, VIC 53127, Australia; Departments of Medicine and Neurology, the Royal Melbourne Hospital, The University of Melbourne, Parkville, VIC 3050, Australia; Department of Neuroscience and Human Genetics, Meyer Children’s Hospital IRCCS, Florence 50139, Italy; Department of Radiology, AOU Cagliari and University of Cagliari, Cagliari 09042, Italy; Department of Neurology, University of California SanFrancisco, San Francisco, CA 94158, USA; Epilepsy and Clinical Neurophysiology Unit, Scientific Institute IRCCS E. Medea, Conegliano 31015, Italy; Children’s National Hospital (CNH), Washington, DC 20010, USA; Department of Neurology, University Hospital Zurich, Zurich 8091, Switzerland; Department of Developmental Neuroscience, IRCCS Foundation Stella Maris, Pisa 56128, Italy; Department of Psychiatry, Neuroscience Institute, University of Cape Town, Cape Town 7925, South Africa; Centre of Excellence in Epilepsy at the Neuro and McConnell Brain Imaging Centre, Montreal Neurological Institute, McGill University, Montreal, QC H3A 2B4, Canada; Department of Neurology and Epileptology, Hertie Institute for Clinical Brain Research, University of Tübingen, Tübingen 72076, Germany; Department of Neurology, CUB Erasme Hospital, Hôpital Universitaire de Bruxelles, Université Libre de Bruxelles, Brussels 1070, Belgium; Department of Neurological Sciences, Rush University Medical Center, Chicago, IL 60612, USA; Department of Biomedical, Metabolic and Neuronal Science, University of Modena and Reggio Emilia, Modena 41125, Italy; Department of Biomedical, Metabolic and Neuronal Science, University of Modena and Reggio Emilia, Modena 41125, Italy; Neurophysiology Unit and Epilepsy Centre, AOU Modena, Modena 41126, Italy; Department of Neurology, School of Medicine at Hofstra/Northwell, Hempstead, NY 11549, USA; Institute of Neurobiology, Universidad Nacional Autónoma de México, Querétaro 76230, Mexico; University of Pittsburgh Comprehensive Epilepsy Center (UPCEC), Department of Neurology, School of Medicine, University of Pittsburgh, Pittsburgh, PA 15213, USA; Department of Neurology, Perelman School of Medicine, University of Pennsylvania, Philadelphia, PA 19104, USA; Center for Neuroengineering and Therapeutics, University of Pennsylvania, Philadelphia, PA 19104, United States; Department of Neurology, David Geffen School of Medicine at UCLA, Los Angeles, CA 90095, USA; Clinic for Neurology, University Medical Center Göttingen, Göttingen 37075, Germany; Department of Neurology, NYU Grossman School of Medicine, NewYork, NY 10016, USA; Florey Institute of Neuroscience and Mental Health, Heidelberg, VIC 3084, Australia; Department of Neurology, NYU Grossman School of Medicine, NewYork, NY 10016, USA; NYU Comprehensive Epilepsy Center, NewYork, NY 10016, USA; Department of Neurology, Langone School of Medicine, NewYork University, New York, NY 10016, USA; Department of Neurology, Emory University, Atlanta, GA 30322, USA; Department of Radiology, Jinling Hospital, Affiliated Hospital of Medical School, Nanjing University, Nanjing 210002, China; Institute of Neurology, Magna Græcia University, Catanzaro 88100, Italy; Neuroscience Research Center, Magna Græcia University, Catanzaro 88100, Italy; Department of Neurology, Medical University of South Carolina, Charleston, SC 29425, USA; Department of Neurology, FCM, University of Campinas—UNICAMP, Campinas, SP 13083-888, Brazil; Brazilian Institute of Neuroscience and Neurotechnology, Campinas, SP 13083-970, Brazil; Imaging Genetics Center, Mark and Mary Stevens Neuroimaging and Informatics Institute, Keck School of Medicine, University of Southern California, Marina del Rey, CA 90292, USA; Department of Clinical and Experimental Epilepsy, UCL Queen Square Institute of Neurology, London WC1N 3BG, UK; Chalfont Centre for Epilepsy, Chalfont St Peter, Bucks SL9 0RJ, UK; Tri-institutional Center for Translational Research in Neuroimaging and Data Science (TReNDS), Georgia State, Georgia Tech, Emory, Atlanta, GA 30303, USA; Department of Neurology, University of South Carolina, Columbia, SC 29203, USA; Department of Radiation Medicine and Applied Sciences, University of California, San Diego, La Jolla, CA 92037, USA; Department of Psychiatry, University of California, San Diego, La Jolla, CA 92037, USA

**Keywords:** epilepsy, diagnosis, lateralization, AI, MRI

## Abstract

Diagnostic MRI evaluation of temporal lobe epilepsy (TLE) depends on the subjective visual interpretation of MRI images. These interpretations could be enhanced by quantitative artificial intelligence (AI) support tools. Humans often make sequential and conditional decisions during their radiological interpretations, such as whether an abnormality is present and, if present, characterizing the abnormality. It is not known whether it is superior to train AI to treat every decision separately in a similar step-wise manner or to train a model holistically on all decisions simultaneously. Here, we analysed three large epilepsy MRI datasets [*n* = 3676, 2320 people with epilepsy and 1356 healthy controls (HC)] to perform two tasks: (i) establish the presence of a TLE pattern on MRI and (ii) determine TLE pattern lateralization. We compared Step-wise models that independently classify TLE versus HC and lateralize patients as left TLE (L-TLE) or right TLE (R-TLE), against a simultaneous model trained to distinguish all three classes in a single step. To do this, 3D volumetric T1-weighted images were input into an EfficientNetV2 model multiple times to ensure reproducibility of results. Class prediction, model classification confidence and saliency maps were output for interpretability. Step-wise models outperformed the Simultaneous model on both tasks (both *P*s < 0.001), with an average ∼2.8% accuracy increase for discriminating HC from TLE and an average 12.7% accuracy increase for distinguishing L-TLE from R-TLE. For both the Step-wise and Simultaneous models, important features discriminating TLE from HC included the known TLE limbic pattern involving the hippocampus, parahippocampal cortical regions, cingulate cortex and lateral temporal regions. However, there was less concordance between the Step-wise and Simultaneous models for the L-TLE versus R-TLE task (all Fisher’s *Z*s > 10.5, *P*s < 0.001); the Step-wise model focused less on subcortical regions such as the thalamus and hippocampus and focused more on distributed cortical pathology. Across the two Step-wise models, 95.1% of TLE patients had accurate classifications in either HC versus TLE and/or L-TLE versus R-TLE tasks. These results included 69.6% of patients being both correctly labelled as TLE and lateralized, 13.9% being correctly labelled TLE but lateralized incorrectly and 11.6% being lateralized correctly but not detected as TLE. These findings provide evidence that diagnostic tasks with simpler, Step-wise AI models may enhance diagnostic performance and interpretability in clinical workflows. Future AI clinical support tools can leverage this step-wise approach in the early identification of TLE-related structural patterns, supporting timely diagnosis and treatment decisions.

## Teaser

Kaestner *et al*. report that dividing diagnostic AI tasks into sequential steps improves accuracy for temporal lobe epilepsy classification. Comparing models across 3676 MRIs, the Step-wise approach provided superior results and distinct anatomical insights into pathological patterns. They conclude that Step-wise AI better mirrors clinical decision-making, enhancing radiological classification.

## Graphical Abstract

**Graphical Abstract:**
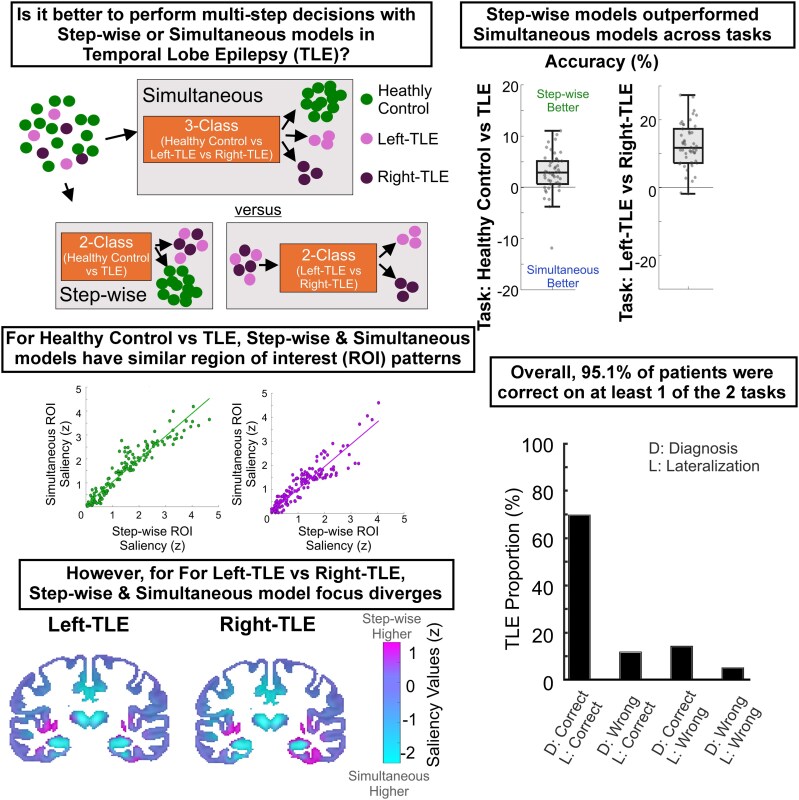

## Introduction

Temporal lobe epilepsy (TLE) is the most common cause of focal epilepsy in adults.^1,2^ A confident diagnosis of TLE depends on the alignment of several factors, including the spatial distribution of brain activity on scalp electroencephalography (EEG), characteristic clinical features (semiology) and MRI abnormalities.^3-6^ The most common qualitative visual MRI marker in TLE is hippocampal atrophy identified on T1-weighted images. Quantitative neuroimaging has additionally revealed a distributed pattern of structural abnormalities in TLE, including limbic atrophy extending beyond the hippocampus. However, this distributed pattern is often subtle and difficult to detect visually, limiting its use in clinical diagnosis. In research settings, artificial intelligence (AI) has leveraged this marker to outperform human experts in detecting and localizing structural correlates of TLE, especially in MRI-negative cases, achieving performance gains of up to ∼30 percentage points.^7-10^ These results highlight the potential value of incorporating AI as a clinician support tool in the radiological evaluation of epilepsy.

A necessary step towards bridging AI integration into clinical practice involves understanding how AI methods and human clinical decision-making do or do not align. A prominent view in diagnostic practice research advocates decision tree approaches, in which sequential, step-wise decisions improve efficiency and reduce errors.^11-13^ This has led to advocacy for implementing simple, interpretable approaches in AI support tools as well.^14^ Therefore, understanding how the efficacy of differing AI approaches to multi-step problems will help humans to better interpret AI outputs, facilitating integration of these outputs into their own decision-making context. A typical neuroradiological read decomposes a complex problem into simpler sub-decisions, whose answers lead to subsequent questions. These decompositions can move from global-to-local, lesion-to-phenotype or pattern-to-aetiology as examples, using a sequential and conditional structure. Therefore, while designing AI models that mimic human radiological reads one-to-one is unlikely, they can mirror the general principles of a sequential and conditional structure. However, AI studies in epilepsy often rely on single-task learning, performing only one task at a time [e.g. distinguishing healthy controls (HC) versus TLE].

Transitioning to multi-step models is complicated by the challenge of finding optimal AI approaches for multi-task problems, which is an area of contention.^15-17^ AI can be configured to approach tasks simultaneously or step-wise; however, it remains unclear which configuration yields superior performance and human interpretability. A step-wise logic reflects both pedagogical tradition and human cognitive tractability in clinical practice. However, AI models are not identical to human cognitive architectures and may benefit from a simultaneous problem formulation, in which all diagnostic dimensions are learned together and mutually inform one another. In this study, we define ‘Step-wise’ models as solving a single task (e.g. detecting TLE) at any one time. In contrast, ‘Simultaneous’ models attempt multiple tasks in a single model by distinguishing among all outcome classes (e.g. HC, L-TLE and R-TLE). Therefore, as AI is being developed and refined for epilepsy, it is crucial to evaluate which method (Step-wise versus Simultaneous) is a better fit for AI radiological support tools.

In this study, we use three large three-dimensional MRI epilepsy datasets^7,18,19^ (*n* = 3676) to perform two critical diagnostic tasks: (i) detection of TLE-related structural signature and (ii) lateralization of these abnormalities to the left or right temporal lobe. We were interested not only in differences in performance but also differences in outputs related to human understanding of AI decision-making such as a model’s confidence in its classifications and visualization.^20-23^ We present data from 26 sites, providing a robust challenge across highly heterogenous datasets. The inherent heterogeneity of scanner acquisition parameters and geographic spread increases confidence in generalizability of results.^10,24^ We ask the following questions: (i) are simpler, Step-wise models more accurate than a Simultaneous model on HC versus TLE and L-TLE versus R-TLE classification? (ii) Are there significant differences between Step-wise and Simultaneous models in interpretability metrics, i.e. decision confidence and visualization output? (iii) If Step-wise models are placed end-to-end in a cascaded manner, what is the overall accuracy? By cascaded, we mean a sequence of individual Step-wise models, where output generated by one model in the sequence serves as the input for the subsequent model. These results not only inform the optimization of AI tools for epilepsy diagnosis but also shed light on how AI decision-making aligns with or diverges from human clinical reasoning which is broadly sequential and conditional.

## Materials and methods

### Participants

#### Data sources

A total of 3676 participants [HC, *n* = 1356; L-TLE, *n* = 1071; R-TLE, *n* = 777; genetic generalized epilepsy (GGE), *n* = 318; frontal lobe epilepsy (FLE), *n* = 154] derived from 26 different imaging sites were available from three datasets: Enhancing NeuroImaging and Genetics through Meta-Analysis-Epilepsy (ENIGMA-Epilepsy, *n* = 2770),^25,26^ Connectome Abnormalities to Predict Epilepsy Surgery (CAPES, *n* = 748),^7^ and Epilepsy Connectome Project (ECP, *n* = 158).^19^ Inclusion criteria across datasets are contained in their respective citations. Broadly, HC patients across datasets were included based on the absence of a major neurological or psychiatric disorder and the absence of a brain structural abnormality (either known a priori or discovered through research scanning). Additionally, for a sensitivity analysis of non-TLE patients with epilepsy, patients from the above datasets GGE and FLE were used. Ethical approval for the study was obtained through the Institutional Review Boards where participants were enrolled by the relevant leads in each dataset. All relevant ethical regulations were followed, and informed consent was obtained at the respective facilities.

#### Sample

Table 1 contains descriptive counts and statistics for HC, L-TLE and R-TLE. Centres varied with regard to readily available demographic and clinical variables, which contributed to variability in the total number of clinical and demographic data points available. Across datasets, patients were included if they were at least 18 years old and had a diagnosis of unilateral drug-resistant TLE as defined by the International League Against Epilepsy.^27^ Bilateral TLE (BTLE) cases were not included to keep analysis limited to tractable, focused problems at this stage. TLE cases included both those clinically identified as having hippocampal sclerosis and those clinically identified as non-lesional. Diagnostic confirmation was made at each site and involved consensus of a multidisciplinary clinical team based on electrophysiology, structural data, semiology and other clinical considerations across the sites. Patients with the presence of other major neurological diseases, mass-occupying lesions or additional identified epileptic foci were excluded from the study.

**Table 1:** Clinical and demographic features of the dataset

	HC	L-TLE	R-TLE	HC versus TLE stats	L-TLE versus R-TLE stats
*N*	1356	1071	777		
Age	34.7 (12)	38.5 (12)	39.1 (12)	*t*(3167) = 9.22; *P* < 0.001, *d* = 0.33	*t*(1845) = −1.05; *P* = 0.30, *d* = −0.05
Sex (female/male)	732/527	613/450	453/313	*χ* ^2^ = 0.01, *P* = 0.94	*χ* ^2^ = 0.40, *P* = 0.53
Handedness (ambi/left/right)	10/32/579	9/72/506	5/36/351	*χ* ^2^ = 16.46; *P* < 0.001	*χ* ^2^ = 2.43; *P* = 0.30
HS (yes/no)		395/366	259/266		*χ* ^2^ = 0.82, *P* = 0.36

HC, healthy control; TLE, temporal lobe epilepsy; F, female; M, male; L, left; R, right; HS, hippocampal sclerosis (i.e. MRI positive or negative).

Categorical variables are listed as *N*. Continuous variables are listed as mean (standard deviation).

### Imaging acquisition

Scanner type and acquisition parameters were heterogeneous across datasets and institutions. Scan parameters for CAPES^7^ and ECP^19^ are available at their respective citations. For ENIGMA, Supplementary Table 1 includes scan parameters.

### Image pre-processing

3D volumetric T1-weighted MRI images were preprocessed using Nii_preprocess (https://github.com/neurolabusc/nii_preprocess) using SPM12 (version 7771; https://www.fil.ion.ucl.ac.uk/spm/software/spm12/) and CAT12 (version 2000; http://www.neuro.uni-jena.de/cat12/).

#### Image processing pipeline

The pipeline included the statistical parametric mapping (SPM) with the CAT12 extension^28^: normalization, tissue segmentation and modulation via the Jacobian determinant. We normalized all T1-weighted images into standard stereotaxic MNI152 space (113 × 137 × 113) using the SPM normalize function with the following parameters: bias regularization = 0.0001, bias full width at half maximum = 60, tissue probability map = TPM.nii, voxel size = 1 × 1 × 1 mm^3^ and fourth-degree b-spline interpolation. Manual registration quality assurance was not performed in the current dataset due to the large volume of scans. Before using the final data, we zero-padded the first and last dimension (i.e. sagittal and axial) to 128 × 137 × 128 for processing efficiency reasons (128 is a power of 2). We also cropped slices in the second (i.e. coronal) direction which included only values at or close to zero at the front and rear of the image since they contained no anatomical information, leaving us with a final image size of 128 × 115 × 128. Harmonization was not applied due to our previous findings that common approaches used in neuroimaging (i.e. ComBat) did not improve model performance.^10^

### Artificial neural network

#### Architecture

**Figure 1:**
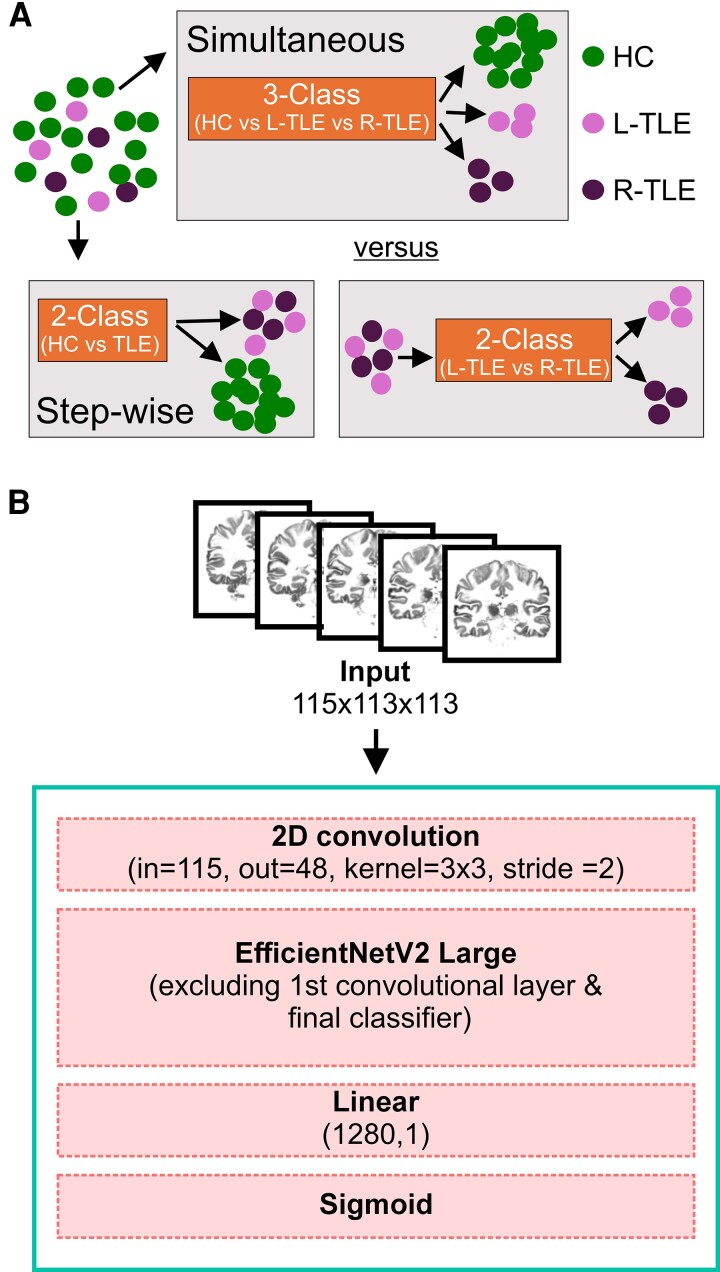
**Study design and convolutional neural network model architecture.** (**A**) Step-wise models employ a sequence of individual, distinct models focused on a specific task. The output could be concatenated in a full cascaded model where output generated by one model in the sequence serves as the input for the subsequent model. This sequential processing allows for a modular approach to complex analytical tasks. Conversely, simultaneous approaches use a single model that is trained to perform multiple related tasks concurrently, and the model is fully aware of all classes during training. (**B**) We adopted EfficientNetV2 for the three classification models, modifying the first convolution layer for 115 input channels (post-cropped coronal MRI) and replacing the final classifier with a linear layer and sigmoid activation for the Step-wise models and a softmax activation for the Simultaneous model. Identical models were trained on HC versus TLE, L-TLE versus R-TLE and HC versus L-TLE versus R-TLE. The final architecture includes a feature extractor with ∼117 million parameters, producing a 1280-dimensional feature vector, and a linear classifier with 1281 parameters, including a bias term. HC, healthy controls; L-TLE, left temporal lobe epilepsy; R-TLE, right temporal lobe epilepsy.

An EfficientNetV2 model for our three classification tasks was adopted^29^ (https://github.com/pytorch/vision/blob/main/torchvision/models/efficientnet.py). EfficientNetV2 was chosen based on a search through models for optimal performance on both two-class and three-class tasks (see Supplementary Table 2). For the EfficientNetV2 model, we accomplished this by replacing the first convolution layer of the architecture by setting the number of input channels to 115 (post-cropped MRI along the coronal axis). For the Step-wise models, we replaced the final classifier with a linear layer that outputs a single neuron followed by a sigmoid activation; for the Simultaneous model, we used a linear layer with three output neurons and a softmax activation. Two identical Step-wise models were built, one for each task: HC versus TLE and L-TLE versus R-TLE. Another identical Simultaneous model was built for performing both tasks at once: HC versus L-TLE versus R-TLE (Fig. 1A). We selected EfficientNetV2 as the final architecture based on benchmark performance across candidate models. The final architecture is depicted in Fig. 1, comprising a feature extractor and classifier. The EfficientNetV2’s feature extractor contains approximately 117 million parameters which outputs a 1280-dimensional feature vector. The classifier is a linear classifier consisting of 1281 parameters (including a bias term).

#### Model training and execution

After each epoch, validation loss was calculated with the best model being saved. A patience of 40 epochs was used. We minimized the binary cross-entropy loss for binary predictions and cross-entropy loss for three-class classification. The model was trained using the Adam optimizer with an initial learning rate of 0.001. A learning rate scheduler named ‘Cosine Annealing with Warm Restarts’ with 30 epochs until the first restart and a minimum learning rate of 5e-5 was used. Random data augmentation techniques such as random flips and rotation were employed to enhance our dataset. We trained models across a total of 10 runs, with each run comprising running across a 5-fold split, resulting in 50 total model instances (10 runs × 5 folds). For each of the 10 runs, the model training, execution, independent testing, and cascaded testing comprised the following parts, each repeated five times for each fold.

##### Initial split

First, we employed stratified 5-fold cross-validation (S5-CV) to divide the data into five equal parts, with a consistent distribution of classes across all folds. Four folds were used for training and 1-fold was selected as the independent test set, which models were blind to during training. The same input seed was used across all models to ensure splits were identical for model comparison purposes.

##### Model training

The four folds (80% of total data) were then split into an 80% training set (64% of total data) and a 20% validation set (16% of the total data), with the remaining 20% of the overall data being held out for independent later testing. There were approximately equal amounts of data from the different sites and datasets spread across the folds. During training, we used a resampling technique to overcome class imbalance problem in our dataset. While creating a batch, we sampled equal numbers of examples from each type of class and formed a batch.

##### Independent testing

For each of the 10 runs, we ran a 5-fold cross-validation in which four folds were used for training and validation, and the fifth, held-out test fold was fed into the model selected from the validation data. This process was repeated five times on each run, so each of the five folds was used as a test fold once. Therefore, 50 trained models were generated (10 runs with five folds), and testing predictions were always derived from unseen data, which were then compared to the true labels. Finally, the chosen evaluation metrics were calculated on these predictions to provide a final, unbiased assessment of the model’s generalizable performance.

##### Cascaded testing

For our final test of how the two Step-wise models perform, we ‘cascaded’ the HC versus TLE and L-TLE versus R-TLE models using the same independent fold, with one important modification (Fig. 1A). The HC versus TLE step was identical, as the seeds kept the test fold identical. The second step was different, as only images that were identified as ‘TLE’ were then fed into the second step of L-TLE versus R-TLE. Therefore, any true TLEs that were misidentified as ‘HC’ were considered to have automatically failed the L-TLE versus R-TLE task as they were never seen by this model.

Hardware: all model training, validation and testing were performed on a single RTX 4090 with 24GB of VRAM.

### Model evaluation

#### Performance metrics

Performance was characterized by accuracy, F1-score and precision–recall AUC (AUC-PR; area under the curve of precision against recall at variable threshold). Accuracy is a simple calculation of (correct cases/total cases). The F1-score combines precision (also called ‘positive predictive value’) and recall (also called ‘sensitivity’) into a single measure via the harmonic mean. It complements accuracy when classes are imbalanced. Finally, we report AUC-PR, which plots precision against recall across multiple thresholds. It focuses on the performance of the model on the positive class. As mentioned, median performance and variability were reported for each model from 50 model instances (10 runs × 5 folds) allowing us to quantify model stability on our performance metrics.

For the Step-wise models, calculating these three metrics was simple, as the model classes and test classes were identical (e.g. the model predicted HC and TLE and was tested on the accuracy of HC and TLE predictions). However, for the Simultaneous model, some modifications were made to ensure a fair comparison.

##### HC versus TLE task

The Simultaneous model was considered to have predicted ‘TLE’ if it predicted either ‘L-TLE’ or ‘R-TLE’, reducing its performance to two classes (i.e. HC versus TLE).

##### L-TLE versus R-TLE task

For this task, ‘HC’ cases were excluded from the test dataset, so its performance was only calculated on ‘all’ ‘TLE’ cases, the same as the Step-wise model. Laterality was then calculated by ‘re-basing’ L-TLE and R-TLE probabilities by ‘TLE probability’. For example, if the Simultaneous model’s probability was 60% HC, 30% L-TLE and 10% R-TLE, this was considered 75% L-TLE and 25% R-TLE for this task [L-TLE = 30%/(30% + 10%) and R-TLE = 10%/(30% + 10%)]. Therefore, even if a patient was overall classified as ‘HC’ by the Simultaneous model, they still received a chance to correctly lateralize when comparing performance on the L-TLE versus R-TLE task.

### Feature visualization

A simple backpropagation method^30,31^ (https://github.com/meta-pytorch/captum) was used to create saliency maps which highlight how strongly voxels contributed to each classification task. The process computes the gradient of the activation of the neuron responsible for the prediction with respect to the input MRI, by performing a backward pass, and then takes the absolute value of that gradient and normalizes it. Therefore, voxels that contribute the most to the classifier have higher saliency values. We only used the final model of each iteration to compute the saliency maps and only for the associated test set samples. Stated another way, saliency was only calculated for each subject when that individual was in the held-out test fold. Saliency values were *z*-scored for ease of interpretation. We used the AAL3 atlas^32^ to derive average region of interest (ROI) saliency values for each subject.

### Statistical analysis

#### Demographics and clinical variables

One-way analysis of variance (ANOVA) for continuous variables or Fisher’s exact tests for categorical variables examined differences among classes.

#### Step-wise and Simultaneous model comparisons

To compare performance between the Step-wise and Simultaneous models across our two tasks, we employed several approaches to ensure robustness of results. First, we computed the frequency distribution comparison index (FDCI) value [(# of times model 1 accuracy > model 2 accuracy/total # of models) × 100 for a percentage]. To make a fair comparison, we performed two comparisons for the independent models. (i) HC versus TLE: for the three-class model, L-TLE and R-TLE were considered correctly diagnosed as TLE. (ii) L-TLE versus R-TLE: for the three-class model, only performance on the L-TLE and R-TLE patients was included (not considering performance on HC at all). We also ran a simple *t*-test to assess effect sizes and to determine whether this approach agreed with the FDCI results.

#### Extra-TLE testing

To test whether our ‘HC versus TLE’ model was specific to TLE or detected epilepsy more generally, we took our dataset of GGE and FLE patients and obtained the model’s prediction in the HC versus TLE model. This indexes whether the model is picking up on general features of seizures (good performance on GGE and FLE), focal epilepsy (good performance on FLE but poor performance on GGE) or only on TLE features (poor performance on GGE and FLE).

#### Single-subject classification confidence

To assess classification confidence at the single-subject level, we extracted the transformed output of the CNN’s final layer, a scalar probability between 0 and 1 representing the model’s predicted likelihood for the target class, and used these per-subject probability scores as our ‘classification confidence’ metric. An average of this classification confidence was created for each subject from the 10 times an individual was in the test fold on each run, re-scaled from 0 (certain wrong class) to 100 (certain right class). To characterize any differences between Step-wise and Simultaneous models on classification confidence, we ran a simple *t*-test.

#### Saliency correlations

To compare saliency value correlation across regions between Simultaneous and Step-wise models, average saliency values were calculated within the AAL3 atlas, and then regions were correlated with Spearman’s rho. To determine any potential differences between correlations, a Fisher’s *Z*-test was used.

## Results

### Patient demographics

Table 1 contains statistical tests between HC versus TLE (i.e. pooled L-TLE and R-TLE) and between L- and R-TLE. Between HC and TLE, both age (*t*(3167) = 9.22; *P* < 0.001 *d* = 0.33) and handedness (*χ*^2^ = 16.46; *P* < 0.001) significantly differed. HC were on average 3.8 years younger than TLE, and TLE had a higher proportion of left-handedness. These differences between the groups were judged to be small and not clinically relevant, with statistical significance driven by the large sample size of this study. Therefore, they were not considered as nuisance variables in the following analyses. There were no significant differences between L- and R-TLE (all *P*s > 0.05).

### Step-wise versus Simultaneous model performance

#### Model performance

**Figure 2:**
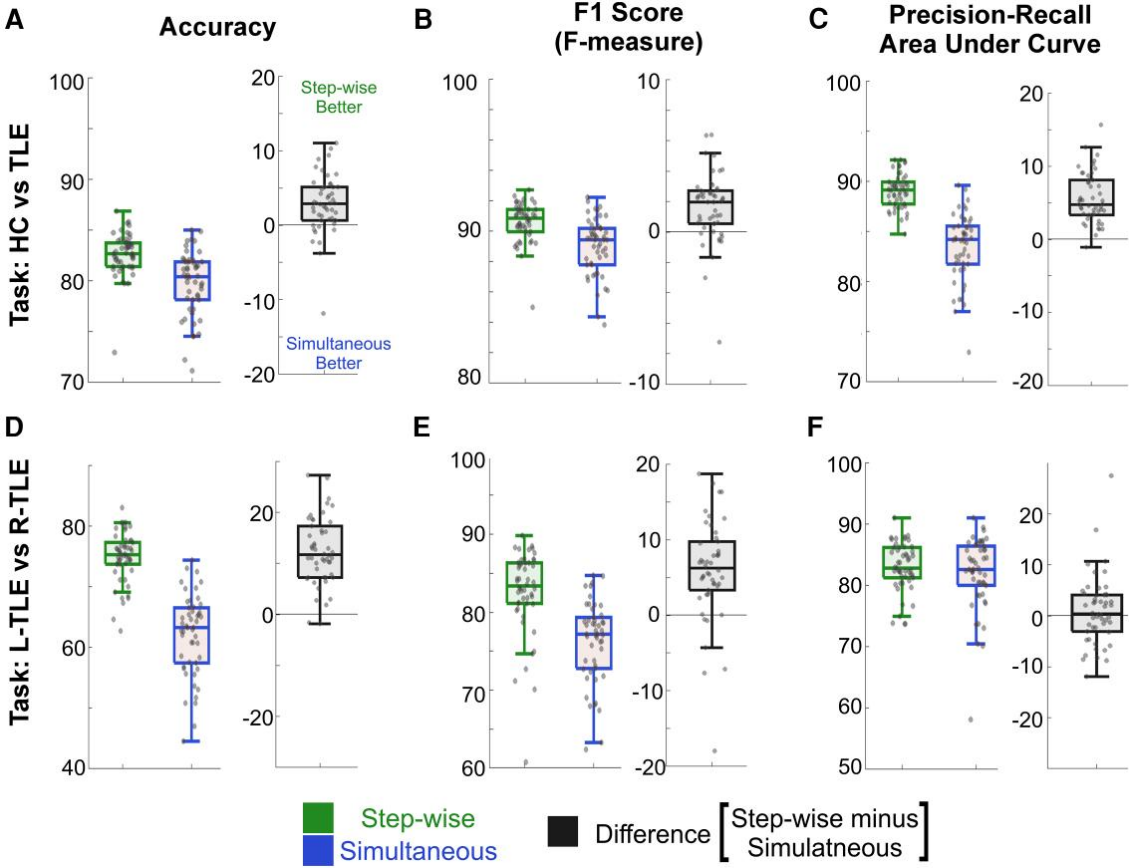
**Average performances comparing Step-wise and Simultaneous models.** (**A**) Accuracy on the HC (*N* = 1356) versus TLE (*N* = 1848) task for the Step-wise (green) and Simultaneous (orange) models showing a 2.8% increase for Step-wise in accuracy (*I*(49) = 5.2, *P* < 0.001). (**B**) F1-score performance which was higher in the Step-wise model (*t*(49) = 4.9, *P* < 0.001). (**C**) Precision–recall area under the curve performance which was higher in the Step-wise (*t*(49) = 12, *P* < 0.001). (**D**) Accuracy performance on the L-TLE (*N* = 1071) versus R-TLE (*N* = 777) task for the Step-wise (green) and Simultaneous (right) models showing a 12.7% increase for Step-wise which was higher (*t*(49) = 13, *P* < 0.001). (**E**) F1-score performance which was higher in the Step-wise model (*t*(49) = 6.3, *P* < 0.001). (**F**) Precision–recall area under the curve performance which was not significantly different (*t*(49) = 1.1, *P* = 0.30). HC, healthy controls; L-TLE, left temporal lobe epilepsy; R-TLE, right temporal lobe epilepsy; F1, F-measure. Each data point in the boxplots represents a single individual (patient or control).

Table 2 presents performance statistics for HC versus TLE and L- versus R-TLE classification based on either the Step-wise (i.e. two-class) or Simultaneous (i.e. three-class) models. Figure 2 illustrates model performance. We found that the Step-wise model significantly outperformed the Simultaneous model on both tasks.

**Table 2:** Task performance comparison of Step-wise and Simultaneous models

Task	Model type	Accuracy	F1	Precision–recall AUC
TLE versus HC	Step-wise (two-class)	82.6 (2.13), 73–86.9	90.6 (1.3), 85–92.7	0.89 (0.02), 0.84–0.92
	Simultaneous (three-class)	79.8 (3.18), 71.1–85	89 (1.88), 83.8–92.2	0.83 (0.03), 0.73–0.90
	Comparison	FDCI: 82% *t*(49) = 5.2, *P* < 0.001	FDCI: 80% *t*(49) = 4.9, *P* < 0.001	FDCI: 98% *t*(49) = 12, *P* < 0.001
L-TLE versus R-TLE	Step-wise (two-class)	74.8 (4.2), 62.7–83	82.6 (5.5), 60.7–89.8	0.83 (0.04), 0.74–0.91
	Simultaneous (three-class)	62.1 (6.7), 44.6–74.3	76.3 (5.2), 62.3–84.7	0.82 (0.06), 0.58–0.91
	Comparison	FDCI: 96% *t*(49) = 13, *P* < 0.001	FDCI: 88% *t*(49) = 6.3, *P* < 0.001	FDCI: 54% *t*(49) = 1.1, *P* = 0.3

All models are EfficientNetV2.

Performance cells: mean (StDev), minimum–maximum.

FDCI, frequency distribution comparison index [(# of times Step-wise > Simultaneous/total # of models) × 100].

For HC versus TLE, the Step-wise model had greater accuracy (FDCI: 82%; *t*(49) = 5.2, *P* < 0.001), F1-score (FDCI: 80%; *t*(49) = 4.9, *P* < 0.001) and AUC-PR (FDCI: 98%; *t*(49) = 12, *P* < 0.001). The average increase in performance was modest, with accuracy on average increasing ∼2.8%, F1-score increasing ∼1.6% and AUC-PR by 0.05.

For L-TLE versus R-TLE, the Step-wise model outperformed the Simultaneous model in accuracy (FDCI: 96%; *t*(49) = 13, *P* < 0.001) and F1-score (FDCI: 88%; *t*(49) = 6.3, *P* < 0.001). The average increase in performance was larger, with accuracy on average increasing ∼12.7% and F1-score increasing ∼6.3%. In contrast, AUC-PR did not significantly differ between models (FDCI: 54%; *t*(49) = 1.1, *P* = 0.30), suggesting comparable precision–recall dynamics despite the accuracy advantage of the Step-wise model.

#### Model classification confidence

**Figure 3:**
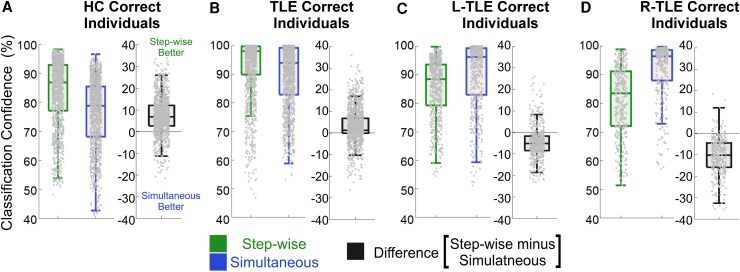
**Model classification confidence when correctly predicting the individual’s class.** (**A**) Average classification confidence on correctly identified HC’s in the HC (*N* = 1356) versus TLE (*N* = 1848) task between the Step-wise and Simultaneous models (left panel) and individual subtractions (right panel; positive means Step-wise model performed better for that individual). Step-wise classification confidence was higher (*t*(2322) = 15.09, *P* < 0.001). (**B**) Average classification confidence on correctly identified TLE’s in the HC versus TLE task between the step-wise and Simultaneous models (left panel) and individual subtractions (right panel). Step-wise classification confidence was higher (*t*(2716) = 7.12; *P* < 0.001). (**C)** Average classification confidence on correctly identified L-TLE in the L-TLE (*N* = 1071) versus R-TLE (*N* = 777) task between the Step-wise (green) and Simultaneous models (left panel) and individual subtractions (right panel). Simultaneous classification confidence was higher (*t*(1220) = −6.07, *P* < 0.001). (**D**) Average classification confidence on correctly identified R-TLE in the L-TLE versus R-TLE task between the Step-wise and Simultaneous models (left panel) and individual subtractions (right panel). Simultaneous classification confidence was higher (*t*(752) = −14.75; *P* < 0.001). HC, healthy controls; L-TLE, left temporal lobe epilepsy; R-TLE, right temporal lobe epilepsy. Each data point in the boxplots represents a single individual (patient or control).

Figure 3 displays model classification confidence among patients and controls correctly classified by both models (i.e. not penalizing average model classification confidence for subjects that either model got wrong). For the HC versus TLE task, the Step-wise model was more certain of HC (*t*(2322) = 15.09, *P* < 0.001, *d* = 0.63) and TLE (*t*(2716) = 7.12; *P* < 0.001; *d* = 0.27) classification. However, for the L-TLE versus R-TLE task, the Step-wise model was less sure than the Simultaneous model for both L-TLE (*t*(1220) = −6.07; *P* < 0.001; *d* = −0.35) and R-TLE (*t*(752) = −14.75; *P* < 0.001; *d* = −1.07).

#### Feature visualization

**Figure 4:**
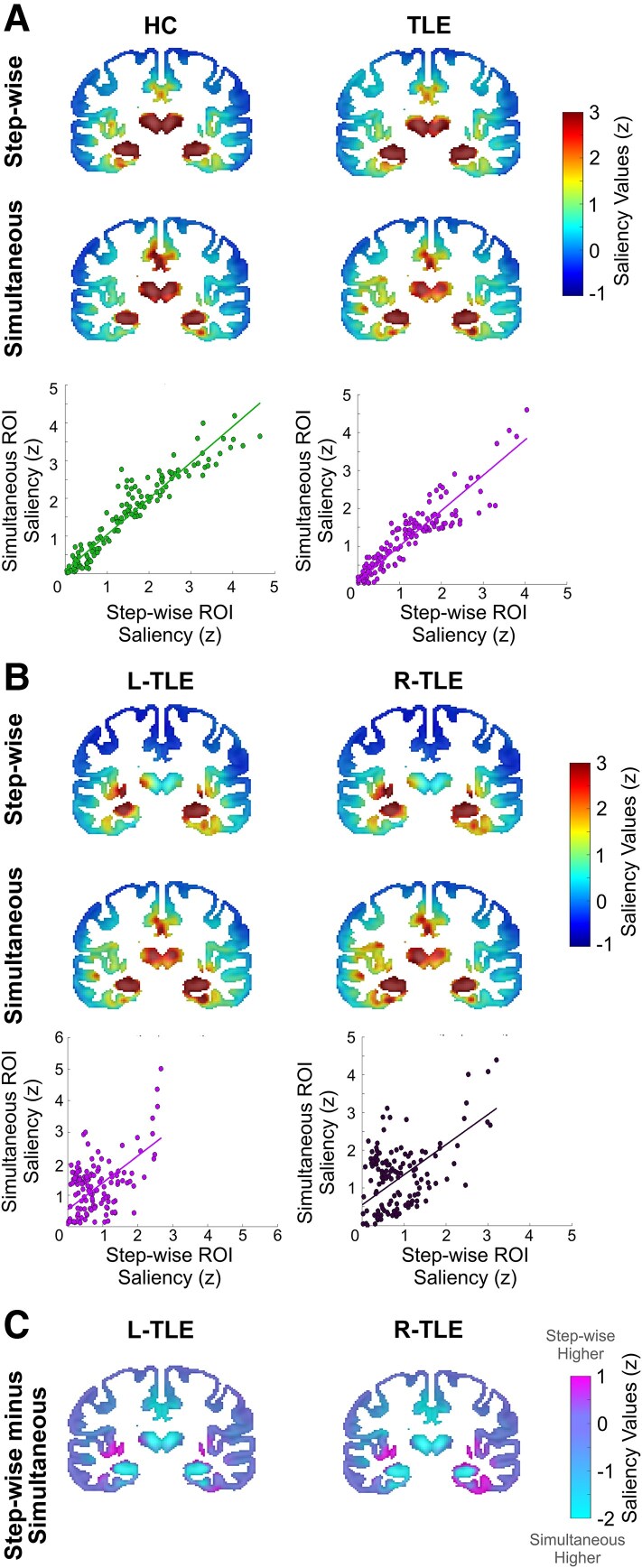
**Saliency maps of focus for the Step-wise and Simultaneous models.** (**A**) HC (*N* = 1356) versus TLE (*N* = 1848) task: nearly identical model foci as shown on the average coronal slice (top) and correlations of average ROI values from the AAL3 atlas. (**B**) L-TLE (*N* = 1071) versus R-TLE (*N* = 777) task: divergent model foci as shown on the average coronal slice (top) and diminished correlations of average ROI values from the AAL3 atlas. We note that both hemispheres’ hippocampi/limbic patterns are highly salient because the model is attending to both, even though the atrophy would be lateralized. This merely reflects that the model compares both hemispheres, not that it believes both hemispheres are equivalent. (**C**) Subtraction brain showing differences between the two models, with Step-wise > Simultaneous values being progressively more pink and Simultaneous > Step-wise values being progressively more blue. In scatterplots, each data point represents an individual from the column (i.e. data points in the HC column scatterplot represent individual HC’s). HC, healthy controls; L-TLE, left temporal lobe epilepsy; R-TLE, right temporal lobe epilepsy; AAL3, Automated Anatomical Labeling Atlas version 3; ROI, region of interest.

Figure 4 illustrates feature visualization similarities and differences across models. At a qualitative level, the Step-wise and Simultaneous models for HC versus TLE were very similar with high salience following a limbic pattern involving the hippocampus, parahippocampal cortical regions, cingulate cortex and some lateral temporal regions. Quantitatively, the correlation was strongly significant (HC salience, rho = 0.97, *P* < 0.001; TLE salience, rho = 0.95, *P* < 0.001).

For the L-TLE versus R-TLE task, the results were more distinct. While areas highlighted were broadly similar to the HC versus TLE task, the Step-wise model focused less on subcortical regions like the thalamus and hippocampus and had a more distributed focus including more cortical regions. Though the correlation was overall still highly significant between Step-wise and Simultaneous models (L-TLE, rho = 0.58, *P* < 0.001, R-TLE, rho = 0.55, *P* < 0.001), all Step-wise correlations in the L-TLE versus R-TLE task were significantly lower than in the HC versus TLE task (all Fisher’s *Z*s > 10.5, *P*s < 0.001).

### Cascaded performance

**Figure 5:**
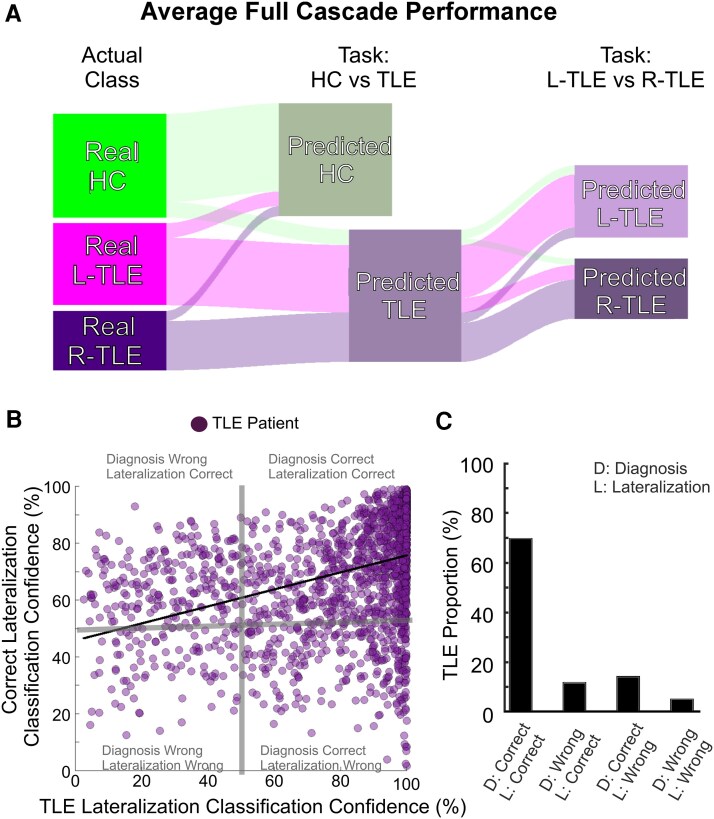
**Fully cascaded performance of our cohort.** (**A**) A Sankey plot showing ultimate patient classification when ‘cascading’ our two Step-wise models sequentially. Cascaded models employ a sequence of individual, distinct models, where output generated by one model in the sequence serves as the input for the subsequent model. Colours from the actual classes (green for Real HC, pink for L-TLE, purple for R-TLE) show propagation of each true class through the two models. (**B**) Plot of the average model classification confidence for each patient averaged across the 10 model runs when the patient was in the test set. Confidences go from 0 (complete confidence in incorrect class) to 100 (complete confidence of correct class). The four quadrants denote the four possible quadrants using an agnostic cut-off of >50% as correct resulting in a 2 × 2 square in which patients could be correct in both tasks, correct only in one task or correct in neither. Each data point is a single patient, with *N* = 1848. (**C**) Illustration of the proportion of patients in each of the four quadrants in B. HC, healthy controls; L-TLE, left temporal lobe epilepsy; R-TLE, right temporal lobe epilepsy.

Figure 5 illustrates performance for patients when cascading across the two Step-wise models. Because the first task (HC versus TLE) exactly matches the results above, we will not repeat them here. For the L-TLE versus R-TLE task, when penalizing the Step-wise model for the misses of the first step in the cascade (i.e. TLE subjects incorrectly labelled HC were judged automatically wrong in the L-TLE versus R-TLE task), the large Step-wise model improvement over the Simultaneous model in the L-TLE versus R-TLE task disappeared (FDCI: 50%; *t*(49) = 0.74; *P* = 0.46). This suggests that nearly the entire improvement of the Step-wise approach model on the L-TLE versus R-TLE task noted above (∼12.7%) was in patients in which the HC versus TLE model fails to correctly classify TLE. Put another way, the observed performance gains of the Step-wise model in lateralization are largely driven by patients incorrectly identified as HC.

To quantify this interpretation, we next took the average model classification confidence for all TLE patients across the 10 runs and correlated the two tasks. We found a significant (rho = 0.58, *P* < 0.001) but not absolute relationship in the classification confidence across tasks. That is, using a neutral 50% model classification confidence cut-off to determine accuracy (i.e. if on average >50% confident of correct class), there are significant proportions of patients that would be correctly determined to be TLE but incorrectly lateralized (13.9%) or incorrectly determined to be HC but correctly lateralized (11.6%). Across the entire sample, only 4.9% of patients would both be incorrectly determined to be HC and also incorrectly lateralized (if they had made it to the second step). Finally, 69.6% were categorized as correct in both tasks using this criterion.

### Extra-TLE performance

To test whether our HC versus TLE model was picking up features general to epilepsy or specific to TLE, we also tested how it classified both GGE and FLE patients. GGE performance was 47.8% ‘correctly’ identified as a patient with epilepsy (i.e. categorized as ‘TLE’ by the model) which is not statistically distinguishable from chance performance (*P* > 0.05). However, performance on FLE patients yielded 86.4% accuracy, which is significantly better than chance (*P* < 0.001). A follow-up *t*-test between probabilities of TLE and FLE patients showed no significant difference in model certainty (*t*(1675) = −0.77, *P* > 0.05).

## Discussion

In this study, we found strong evidence from a large (*n* = 3676) and heterogenous (26 sites) structural neuroimaging sample that AI models perform better on radiological reads in epilepsy when they perform simpler tasks sequentially. With respect to our hypotheses, AI models that are asked to identify whether an MRI belongs to a patient or control (classification) and then to determine the side of the seizure focus (lateralization) perform better on these tasks than a model asked to make both decisions simultaneously. In particular, the better ability of the Step-wise model to lateralize the seizure focus appeared to reflect its attention to broad, subtler cortical abnormalities rather than more prominent subcortical regions. Ultimately, 95.1% of the TLE patients were either correctly diagnosed or had correct lateralization classification (69.6% had both). Therefore, this points towards the need to develop approaches that integrate information across multiple tasks to leverage the sometimes distinct signatures available at each step.

### Step-wise versus Simultaneous models

Medical imaging plays a pivotal role in diagnosis, treatment planning and the ongoing monitoring of patient conditions in modern healthcare. A rapidly increasing volume and variety of medical imaging data is making automated analysis techniques to assist clinicians a necessity. Pipelines, which proceed from initial detection of an anomaly followed by ever more detailed classification, present an appealing approach. Two such approaches have gained prominence: Step-wise models and multi-task (i.e. Simultaneous) models. Step-wise models employ a sequence of individual, distinct models, where output generated by one model in the sequence serves as the input for the subsequent model (i.e. a cascade). This step-wise processing allows for a modular approach to complex analytical tasks. This modular nature offers substantial practical advantages in terms of system flexibility, maintenance and future adaptability. Conversely, simultaneous approaches use a single model that is trained to perform multiple related tasks concurrently. The principle is to leverage shared representations and correlations across the different tasks, improving the learning and performance on each individual task through diverse training challenges. Currently, it is unclear if one approach is superior to the other, and in the absence of strong evidence to favour one or the other, the optimal choice may depend on the specific tasks being addressed. However, we note that often in clinical practice diagnostic hypotheses are iteratively formulated and revised, with subsequent questions guided by the evolving evidence. Such modular and step-wise approaches are currently being developed in the AI diagnostic space as well, lending weight to a focus on building Step-wise models to support this movement.^33^

In terms of raw performance, in our current study the Step-wise models stably outperformed the Simultaneous counterparts on both tasks. This was significant on the HC versus TLE task, though the observed average ∼2.7% improvement was modest. In the L-TLE versus R-TLE task, the improvement was larger, as the observed jump in performance was an average ∼12.7% improvement. We interpret this as strong evidence supporting the implementation of simpler, task-specific models emerging by allowing models to focus on simpler tasks.

The only measure for which Step-wise and Simultaneous models were equivalent was AUC-PR (precision–recall area under the curve, measuring precision against recall at variable thresholds) for the L-TLE versus R-TLE task. This is related to model classification confidence on the lateralization task. In addition to performance, model interpretability is critical for clinical implementation. This includes model classification confidence (discussed here) as well as model visualization (discussed in the next section). In the HC versus TLE task, the Step-wise model was more confident when correctly classifying both HC (*d* = 0.63) and TLE (*d* = 0.27) than the Simultaneous model. The Step-wise model was not only more correct, but when it was correct, it demonstrated higher classification confidence of its accuracy. In contrast, the Simultaneous model showed higher classification confidence for L-TLE (*d* = −0.35) and R-TLE (*d* = −1.07). Here, a more negative effect size (i.e. more negative *d*) means that the Simultaneous model had higher classification confidence. Therefore, in the lateralization task, full access to shared representations and inherent correlations amongst classes appeared to increase classification confidence even though it decreased performance.

### Why Step-wise models outperform Simultaneous models

A noteworthy pattern that may explain the striking difference in performance between the Step-wise and Simultaneous model in the L-TLE and R-TLE task is the difference in model saliency maps. Both model approaches focused on broadly similar but ultimately distinguishable regional patterns. Decades of quantitative neuroimaging research have established that TLE exhibits subtle but specific grey matter abnormalities in both the hippocampus and beyond. Voxel-based morphometry and cortical thickness studies consistently demonstrate that TLE is associated with limbic atrophy involving the entorhinal and perirhinal cortices, anterior nuclei of the thalamus, anterior cingulate, insula and lateral temporal neocortex.^26,34-37^ This pattern is apparent in Fig. 4A, as both model approaches leverage these known regions to determine HC versus TLE. Importantly, both the Step-wise and Simultaneous model for this task had nearly identical foci, evident at both a qualitative and quantitative level. This, along with the modest performance benefit for the Step-wise model, suggests that giving the Simultaneous model access to laterality labels did not modify, or improve, its understanding of the TLE signature relative to the simpler HC versus TLE training labels.

The more intriguing comparison emerges between Step-wise and Simultaneous models when discussing the L-TLE versus R-TLE task. The large performance benefit for the Step-wise model was accompanied by a significant decrease in agreement on areas of neurobiological salience (all Fisher’s *Z* > 10.5, *P*s < 0.001; Fig. 4B). Strikingly, the differences (Fig. 4C) included a decrease on the most important subcortical areas from the HC versus TLE task including the hippocampus and thalamus and a focus on more distributed cortical regions. In general, the Step-wise model had less focus on any one region (i.e. the z-scores were overall lower, especially in high-salience Simultaneous model areas), showing a tendency to distribute attention more broadly, presumably in search of subtle patterns for difficult-to-lateralize patients. We interpret this as a concession from the model that damage to subcortical structures such as the bilateral thalami is largely shared across both L- and R-TLE, and the large number of non-lesional patients in our sample reduced the importance of the hippocampi as it would have reduced salience for this portion of patients. In the future, detection of such subtle and distributed signals, long documented in quantitative neuroimaging, could become a task well-suited for AI tools assisting clinicians.

### The full cascade: avenues of improvement and clinical integration

AI shows great promise in research settings in diagnosis, classification and localization tasks related to epilepsy neuroimaging. However, clinical adoption of AI-assisted tools requires not just promising research results, but translational outputs that are both human interpretable, as discussed above, and reliable and meaningful in the clinical context. Hence, we asked, given the improvements in individual tasks observed with the Step-wise model, how the full cascade performed (Fig. 5A). The slight (∼2.7%) improvement in classification (i.e. HC versus TLE) remained, as this step is identical to the earlier comparison. However, nearly the entire ∼12.7% improvement in the lateralization task by the Step-wise model (i.e. L-TLE versus R-TLE) disappeared, as those subjects were misclassified in the first HC versus TLE classification stage. This is interpretable because these patients were subtle cases, mis-lateralized by the Simultaneous model as well and only detectable by a simpler model with a distributed focus. Therefore, the subtlety would be lost by a model, such as the HC versus TLE Step-wise and Simultaneous models, with a strong focus only on specific high-salience regions.

To investigate this discrepancy in signal between models further, we assessed whether individual patients had stable, discernable signal across runs across the two tasks (i.e. whether a patient would have been correct on average across the 10 runs based on model classification confidence). Comparing model classification confidence across the two tasks had a significant correlation (rho = 0.58, *P* < 0.001; Fig. 5B). We found that the largest proportion of patients had discernable signal in both tasks (69.6%), but unexpectedly, significant proportions were correct on either HC versus TLE (13.9%) or L-TLE versus R-TLE (11.6%) but not the other. Viewed from one perspective, this is very encouraging that a usable signal existed in 95.1% of all TLE patients. But to be viewed as a reliable assistant, AI must make this preliminary 95.1% number accurate across both questions. As each Step-wise model adopted a different strategy, with a high focus on salient regions in diagnosis or a distributed focus in lateralization, they differ and have complementary strengths. Therefore, we argue that the development of meta-models and mutual learning, which combine insights from different sub-models focused on a specific task, could be invaluable in leveraging the complementary model strengths. This further highlights the strengths of cascaded over simultaneous approaches; a modular approach allows investigation at each step of the clinician decision-making process, with insights guiding further development.

A final test of our model was an investigation into whether the ‘TLE’ signal in the ‘TLE versus HC’ model was specific to TLE or could be picking up on more general epilepsy-related signal. To achieve this, we tested whether our model would categorize GGE patients and FLE patients as ‘TLE’ or ‘HC’ (i.e. whether the ‘TLE’ in the model was specific to TLE or also categorized other epilepsy syndromes as ‘TLE’). We found mixed results which we believe illustrate the importance of cascaded neuroradiological AI. GGE was categorized at chance level, suggesting that the signal derived from TLE by the model does not generalize to GGE, which would need other models, and potentially neuroimaging modalities to detect. However, FLE was categorized at essentially the same confidence and accuracy as TLE, suggesting that the HC versus TLE model was deriving a signal generally applicable within focal epilepsies. We believe this necessitates the future development of a two-class model distinguishing focal epilepsies such as FLE and TLE, i.e. additional sequential and conditional steps in the cascade.

### Limitations

A key limitation of any CNN that aspires to be generalizable is the need for harmonization that can be deployed across clinical environments, including different scanners, sequences and patient populations. We present data from 26 sites, providing a robust challenge and indexing current model limitations in performance in highly heterogenous datasets. Previously we found that a widely used feature-based imaging tool for harmonization, ComBat,^25,38^ did not meaningfully improve CNN performance. Therefore, we are still in the process of identifying an appropriate novel method for CNN-based work in TLE.^39,40^ Another approach we could have used in evaluation would be to keep a site withheld for independent evaluation of our models. Further limitations include incomplete clinical and demographic data. ‘Big’ datasets may not be both large and well-characterized; here we chose to analyse a large and heterogenous dataset, due to sample sizes increasing performance and increasing confidence in generalizability.^10,24^ Another issue with ‘Big’ datasets is the impracticality of applying manual quality control across a large volume of scans. For instance, registration in neuroimaging is not a solved problem, and failures will reduce model performance. Therefore, future studies may incorporate QC metrics into model training and testing to see if this improves as well as addresses the real-world problem of clinical scans with varying degrees of quality.

Relatedly, another final weakness is the difficulty to be ‘sure’ of an accurate TLE diagnosis.^8^ In this sample, diagnosis is based on the consensus of a multidisciplinary clinical team based on electrophysiology, structural data, semiology and other clinical considerations across the sites. The gold standard is a patient with a seizure-free outcome following surgery, though this radically decreases the available data points. In an earlier report, we compared performance on confirmed seizure-free TLE and non-seizure-free patients and found similar though improved performance on confirmed TLE,^7^ suggesting that these large datasets may include a small portion of misclassified cases. Further, in this cohort we excluded BTLE to focus our analysis on a tractable set of tasks and model comparisons. However, application of any diagnostic aid tool would occur before BTLE had been ruled out, so future efforts can focus on understanding just how specific this neural pattern is to TLE.^41^ Finally, while this work explores a specific multi-task framework, it’s worth noting that only one such approach has been tested in this study. Future research could delve into alternative multi-task learning methodologies to further understand their potential and limitations in this context.

## Conclusion

In conclusion, we demonstrate the capabilities of CNNs in finding subtle but relevant information for disease diagnosis and classification in one of the largest imaging datasets of epilepsy patients to date. Our findings highlight how simpler models can be more sensitive to the subtleties of each task, which in turn has implication in designing AI radiological support tools. The development of meta-models combining information across tasks and different patient types will be necessary to allow mutual learning of the patterns specific to each patient to fully harness available information. While promising, these developing models will complement—not replace—clinical expertise, EEG interpretation and genetic testing in forming a comprehensive diagnostic picture.

## Supplementary Material

fcag253_Supplementary_Data

## Data Availability

Data are available from the corresponding author from the ENIGMA-Epilepsy dataset upon reasonable request and with appropriate data-sharing agreements. Data from ECP and CAPES are available from their respective cohort PIs upon reasonable request. The code used for processing the images, modelling and visualization is noted in their respective sections.

## References

[1] Wiebe S . Epidemiology of temporal lobe epilepsy. Can J Neurol Sci. 2000;27(S1):S6–S10. 10830320 10.1017/s0317167100000561

[2] Téllez-Zenteno JF, Hernández-Ronquillo L. A review of the epidemiology of temporal lobe epilepsy. Epilepsy Res Treat. 2012;2012:630853. 22957234 10.1155/2012/630853 PMC3420432

[3] Jack CR, Sharbrough FW, Twomey CK, et al Temporal lobe seizures: Lateralization with MR volume measurements of the hippocampal formation. Radiology. 1990;175(2):423–429. 2183282 10.1148/radiology.175.2.2183282

[4] Jobst BC, Cascino GD. Resective epilepsy surgery for drug-resistant focal epilepsy: A review. JAMA. 2015;313(3):285–293. 25602999 10.1001/jama.2014.17426

[5] Foldvary N, Klem G, Hammel J, Bingaman W, Najm I, Lüders H. The localizing value of ictal EEG in focal epilepsy. Neurology. 2001;57(11):2022–2028. 11739820 10.1212/wnl.57.11.2022

[6] Fisher RS, Acevedo C, Arzimanoglou A, et al ILAE official report: A practical clinical definition of epilepsy. Epilepsia. 2014;55(4):475–482. 24730690 10.1111/epi.12550

[7] Gleichgerrcht E, Kaestner E, Hassanzadeh R, et al Redefining diagnostic lesional status in temporal lobe epilepsy with artificial intelligence. Brain. 2025;148:2189–2200. 39842945 10.1093/brain/awaf020 PMC12129735

[8] Gleichgerrcht E, Munsell B, Keller SS, et al Radiological identification of temporal lobe epilepsy using artificial intelligence: A feasibility study. Brain Commun. 2022;4(2):fcab284. 35243343 10.1093/braincomms/fcab284 PMC8887904

[9] Kaestner E, Rao J, Chang AJ, et al Convolutional neural network algorithm to determine lateralization of seizure onset in patients with epilepsy: A proof-of-principle study. Neurology. 2023;101(3):e324–e335. 37202160 10.1212/WNL.0000000000207411 PMC10382265

[10] Kaestner E, Hassanzadeh R, Gleichgerrcht E, et al Adding the third dimension: 3D convolutional neural network diagnosis of temporal lobe epilepsy. Brain Commun. 2024;6(5):fcae346. 39474046 10.1093/braincomms/fcae346 PMC11520928

[11] Banerjee M, Reynolds E, Andersson HB, Nallamothu BK. Tree-based analysis: A practical approach to create clinical decision-making tools. Circ Cardiovasc Qual Outcomes. 2019;12(5):e004879. 31043064 10.1161/CIRCOUTCOMES.118.004879 PMC6555420

[12] Sutton RT, Pincock D, Baumgart DC, Sadowski DC, Fedorak RN, Kroeker KI. An overview of clinical decision support systems: Benefits, risks, and strategies for success. NPJ Digit Med. 2020;3(1):17. 32047862 10.1038/s41746-020-0221-y PMC7005290

[13] Sone D, Beheshti I. Clinical application of machine learning models for brain imaging in epilepsy: A review. Front Neurosci. 2021;15:684825. 34239413 10.3389/fnins.2021.684825 PMC8258163

[14] Rudin C . Stop explaining black box machine learning models for high stakes decisions and use interpretable models instead. Nat Mach Intell. 2019;1(5):206–215. 35603010 10.1038/s42256-019-0048-x PMC9122117

[15] Kamiri J, Wambugu GM, Oirere AM. Multi-task deep learning in medical image processing: A systematic review. International Journal of Computing Sciences Research. 2025; 9:3418–3440. Accessed June 6, 2025. https://repository.mut.ac.ke/xmlui/handle/123456789/6502.

[16] Zhao Y, Wang X, Che T, Bao G, Li S. Multi-task deep learning for medical image computing and analysis: A review. Comput Biol Med. 2023;153:106496. 36634599 10.1016/j.compbiomed.2022.106496

[17] Karar ME, Hemdan EED, Shouman MA. Cascaded deep learning classifiers for computer-aided diagnosis of COVID-19 and pneumonia diseases in X-ray scans. Complex Intell Syst. 2021;7(1):235–247. 10.1007/s40747-020-00199-4 PMC7507595 34777953

[18] Sisodiya SM, Whelan CD, Hatton SN, et al The ENIGMA-epilepsy working group: Mapping disease from large data sets. Hum Brain Mapp. 2022;43(1):113–128. 10.1002/hbm.25037 PMC8675408 32468614

[19] Cook CJ, Hwang G, Mathis J, et al Effective connectivity within the default mode network in left temporal lobe epilepsy: Findings from the Epilepsy Connectome Project. Brain Connect. 2019;9(2):174–183. 30398367 10.1089/brain.2018.0600 PMC6444922

[20] Ellis CA, Miller RL, Calhoun VD. Towards greater neuroimaging classification transparency via the integration of explainability methods and confidence estimation approaches. Inform Med Unlocked. 2023;37:101176. 37035832 10.1016/j.imu.2023.101176 PMC10078989

[21] Rahman MM, Calhoun VD, Plis SM. Looking deeper into interpretable deep learning in neuroimaging: a comprehensive survey. arXiv. [Preprint]. Preprint posted online July 14, 2023:arXiv:2307.09615. Accessed February 16, 2024. doi:10.48550/arXiv.2307.09615

[22] Rahman MM, Mahmood U, Lewis N, et al Interpreting models interpreting brain dynamics. Sci Rep. 2022;12(1):12023. 35864279 10.1038/s41598-022-15539-2 PMC9304350

[23] The Precise4Q consortium, Amann J, Blasimme A, Vayena E, Frey D, Madai VI. Explainability for artificial intelligence in healthcare: A multidisciplinary perspective. BMC Med Inform Decis Mak. 2020;20(1):310. 33256715 10.1186/s12911-020-01332-6 PMC7706019

[24] Abrol A, Fu Z, Salman M, et al Deep learning encodes robust discriminative neuroimaging representations to outperform standard machine learning. Nat Commun. 2021;12(1):353. 33441557 10.1038/s41467-020-20655-6 PMC7806588

[25] Hatton SN, Huynh KH, Bonilha L, et al White matter abnormalities across different epilepsy syndromes in adults: An ENIGMA-epilepsy study. Brain. 2020;143(8):2454–2473. 32814957 10.1093/brain/awaa200 PMC7567169

[26] Larivière S, Rodríguez-Cruces R, Royer J, et al Network-based atrophy modelling in the common epilepsies: A worldwide ENIGMA study. Sci Adv. 2020;6(47):eabc6457. 33208365 10.1126/sciadv.abc6457 PMC7673818

[27] Fisher RS, Cross JH, French JA, et al Operational classification of seizure types by the international league against epilepsy: Position paper of the ILAE commission for classification and terminology. Epilepsia. 2017;58(4):522–530. 28276060 10.1111/epi.13670

[28] Gaser C, Dahnke R. CAT-a computational anatomy toolbox for the analysis of structural MRI data. Hbm. 2016;2016:336–348. 10.1093/gigascience/giae049 PMC11299546 39102518

[29] Tan M, Le QV. EfficientNetV2: Smaller models and faster training. arXiv. [Preprint]. Preprint posted online June 23, 2021:arXiv:2104.00298. doi:10.48550/arXiv.2104.00298

[30] Simonyan K, Vedaldi A, Zisserman A. Deep inside convolutional networks: Visualising image classification models and saliency maps. arXiv. [Preprint]. Preprint posted online April 19, 2014:arXiv:1312.6034. doi:10.48550/arXiv.1312.6034

[31] Kokhlikyan N, Miglani V, Martin M, et al Captum: A unified and generic model interpretability library for PyTorch. arXiv. [Preprint]. Preprint posted online September 16, 2020:arXiv:2009.07896. doi:10.48550/arXiv.2009.07896

[32] Revell AY, Silva AB, Arnold TC, et al A framework for brain atlases: Lessons from seizure dynamics. Neuroimage. 2022;254:118986. doi:10.1016/j.neuroimage.2022.118986 35339683 10.1016/j.neuroimage.2022.118986 PMC9342687

[33] Nori H, Daswani M, Kelly C, et al Sequential diagnosis with language models. arXiv. [Preprint]. Preprint posted online July 2, 2025:arXiv:2506.22405. doi:10.48550/arXiv.2506.22405

[34] Whelan CD, Altmann A, Botía JA, et al Structural brain abnormalities in the common epilepsies assessed in a worldwide ENIGMA study. Brain. 2018;141(2):391–408. 29365066 10.1093/brain/awx341 PMC5837616

[35] Bonilha L, Rorden C, Castellano G, et al Voxel-based morphometry reveals gray matter network atrophy in refractory medial temporal lobe epilepsy. Arch Neurol. 2004;61(9):1379–1384. 15364683 10.1001/archneur.61.9.1379

[36] McDonald CR, Hagler Jr DJ, Ahmadi ME, et al Regional neocortical thinning in mesial temporal lobe epilepsy. Epilepsia. 2008;49(5):794–803. 18266751 10.1111/j.1528-1167.2008.01539.x

[37] Bernasconi N, Duchesne S, Janke A, Lerch J, Collins DL, Bernasconi A. Whole-brain voxel-based statistical analysis of gray matter and white matter in temporal lobe epilepsy. NeuroImage. 2004;23(2):717–723. 15488421 10.1016/j.neuroimage.2004.06.015

[38] Fortin JP, Cullen N, Sheline YI, et al Harmonization of cortical thickness measurements across scanners and sites. NeuroImage. 2018;167:104–120. 29155184 10.1016/j.neuroimage.2017.11.024 PMC5845848

[39] Sinha S, Thomopoulos SI, Lam P, Muir A, Thompson PM. Alzheimer’s disease classification accuracy is improved by MRI harmonization based on attention-guided generative adversarial networks. In: *17th International Symposium on Medical Information Processing and Analysis*. Vol 12088. Campinas, Brazil. SPIE. 2021:180-189. 10.1117/12.2606155 PMC8952312 35340753

[40] Dinsdale NK, Jenkinson M, Namburete AIL. Deep learning-based unlearning of dataset bias for MRI harmonisation and confound removal. NeuroImage. 2021;228:117689. 33385551 10.1016/j.neuroimage.2020.117689 PMC7903160

[41] Chang AJ, Roth R, Bougioukli E, et al MRI-based deep learning can discriminate between temporal lobe epilepsy, Alzheimer’s disease, and healthy controls. Commun Med. 2023;3(1):33. 36849746 10.1038/s43856-023-00262-4 PMC9970972

