# Supplementary material for: Disease detection and classification in temporal lobe epilepsy: step-wise versus simultaneous AI decision models in a multisite neuroimaging study
Source: Brain Commun. 2026 Jun 29;8(4):fcag253. doi: 10.1093/braincomms/fcag253 (PMC13421366; doi:10.1093/braincomms/fcag253)
Supplement: fcag253_Supplementary_Data [file fcag253_supplementary_data.docx]

**Supplementary Table 1.** ENIGMA Site Scan Parameters

| **Centre** | **Scanner manufacturer and model** | **Sequence** | **Acquisition Direction** | **# of Slices** | **Spacing Between Slices** | **Voxel Size (mm3)** | **TI** | **TE** | **TR** | **Flip**  **Angle** |
| --- | --- | --- | --- | --- | --- | --- | --- | --- | --- | --- |
| **CNH** | **DISCOVERY MR750** | **MPRAGE** | **sagittal** | **176** | **0 mm** | **1 x 1 x 1 mm** | **1100 ms** | **3.55 ms** | **2530 ms** | **7** |
| **Emory** | **Prisma_fit** | **MPRAGE** | **sagittal** | **176** | **0 mm** | **1 x 1 x 1 mm** | **1100 ms** | **3.02 ms** | **2300 ms** | **8** |
| **EPICZ** | **DISCOVERY MR750** | **BRAVO** | **sagittal** | **256** | **0.5 mm** | **0.5 x 1 x 1 mm** | **650 ms** | **3.668 ms** | **9.164 ms** | **12** |
| **FSM** | **SIGNA Premier** | **BRAVO** | **sagittal** | **174** | **0.9 mm** | **0.9 x 0.45 x 0.45 mm** | **500 ms** | **3.036 ms** | **7.34 ms** | **8** |
| **Gottingen** | **Prisma_fit** | **TFL3D** | **sagittal** | **176** | **0 mm** | **1 x 1 x 1 mm** | **900 ms** | **3.3 ms** | **2250 ms** | **9** |
| **GWU** | **Skyra** | **MPRAGE** | **axial** | **176** | **0 mm** | **0.9 x 0.9 x 1mm** | **900 ms** | **2.41 ms** | **1800 ms** | **8** |
| **LNF** | **Achieva** | **MPRAGE** | **sagittal** | **192** | **0 mm** | **0.9 x 0.98 x 0.98 mm** | **900 ms** | **2.49 ms** | **1900 ms** | **9** |
| **MNI** | **Prisma_fit** | **MPRAGE** | **sagittal** | **224** | **0 mm** | **0.8 x 0.8 x 0.8 mm** | **900 ms** | **3.14 ms** | **2300 ms** | **9** |
| **MUSC** | **Skyra** | **MPRAGE** | **sagittal** | **192** | **0 mm** | **1 x 1 x 1 mm** | **925 ms** | **4.15 ms** | **2250 ms** | **9** |
| **Nanjing** | **TrioTim** | **MPRAGE** | **sagittal** | **176** | **0 mm** | **1 x 0.5 x 0.5 mm** |  | **2.98 ms** | **2300 ms** | **9** |
| **NYU** | **Allegra** | **MPRAGE** | **sagittal** | **128** | **0 mm** | **1.3 x 1 x 1 mm** | **1100 ms** | **3.25 ms** | **2530 ms** | **7** |
| **Rush** | **DicomCleaner, Verio** | **MPRAGE** | **coronal** | **448** | **0 mm** | **0.43 x 0.6 x 0.43 mm** | **900 ms** | **3.29 ms** | **2300 ms** | **8** |
| **UCL** |  |  | **sagittal** | **256** | **0 mm** | **0.9 x 0.9 x 1 mm** |  |  | **1000 ms** |  |
| **UCLA** | **Prisma_fit** | **MPRAGE** | **axial** | **176** | **0 mm** | **1 x 1 x 1 mm** |  | **2.81 ms** | **110 ms** | **20** |
| **UCSD** | **DISCOVERY MR750** | **FSPGR** | **sagittal** | **172** | **1.2 mm** | **1.2 x 1 x 1 mm** | **600 ms** | **3.156 ms** | **8.1 ms** | **8** |
| **UCSF** | **DISCOVERY MR750** | **3DT1_GRE** | **axial** | **256** | **1 mm** | **1 x 1 x 1 mm** | **1000 ms** | **2.948 ms** | **2750.4 ms** | **9** |
| **UCT** | **Allegra** | **MPRAGE** | **axial** | **208** | **0 mm** | **0.81 x 0.81 x 0.9 mm** |  |  | **2000 ms** |  |
| **ULB** | **Achieva** | **3DT1_TFE** | **sagittal** | **130** | **1.2 mm** | **1.2 x 0.82 x 0.82 mm** |  | **4.178 ms** | **8.79 ms** | **8** |
| **UNAM** | **Achieva** | **T13D_SENSE** | **axial** | **176** | **0 mm** | **1 x 1 x 1 mm** |  | **3.68 ms** | **8.045 ms** |  |
| **UniCa** | **Achieva dStream** | **3DT1** | **axial** | **150** | **1 mm** | **1 x 1 x 1 mm** | **900 ms** | **2.7 ms** | **5.9 ms** | **10** |
| **UNICAMP** | **Achieva** | **T13D_SENSE** | **sagittal** | **180** | **1 mm** | **1 x 1 x 1 mm** |  | **3.201 ms** | **7 ms** | **8** |
| **UniMore** | **Achieva** | **3DT1_TFE** | **axial** | **170** | **0 mm** | **1 x 1 x 1 mm** |  | **4.6 ms** | **9.904 ms** |  |
| **UPenn** | **Prisma_fit** | **MPRAGE** | **sagittal** | **208** | **0 mm** | **0.8 x 0.8 x 0.8 mm** | **1060 ms** | **2.24 ms** | **2400 ms** | **8** |
| **USZ** | **Skyra** |  | **axial** | **252** | **0 mm** | **0.49 x 0.49 x 1 mm** | **904 ms** | **2.54 ms** | **1790 ms** | **9** |

**Supplementary Table 2.** Model Selection based on accuracy on a 2-class and a 3-class model to ensure that the same model performed best across bivariate & multiclass models.

|  | **HC versus TLE**  **Accuracy** | **HC versus L-TLE versus R-TLE**  **Accuracy** |
| --- | --- | --- |
| EfficientNetV2 | 82.9% | 72.9% |
| Resnet18 | 80.6% | 69.2% |
| Coatnet | 78.9% | 63.9% |
| Resnet34 | 80.3% | 71.2% |
| FCNet | 79.5% | 69.2% |
| DSconv | 79.9% | 70.9% |
| Resnt18: https://arxiv.org/abs/1512.03385  Coatnet: https://arxiv.org/abs/2106.04803  Resnet34: https://arxiv.org/abs/1512.03385  FCNet: https://academic.oup.com/braincomms/article/6/5/fcae346/7817881  DSconv: https://arxiv.org/abs/1610.02357  HC: Healthy Controls, L-TLE: Left Temporal Lobe Epilepsy, R-TLE: Right Temporal Lobe Epilepsy | | |
